# Benchmarked performance charts using principal components analysis to improve the effectiveness of feedback for audit data in HIV care

**DOI:** 10.1186/s12913-017-2426-6

**Published:** 2017-07-24

**Authors:** Skevi Michael, Mark Gompels, Caroline Sabin, Hilary Curtis, Margaret T. May

**Affiliations:** 1 School of Mathematics, University of Bristol, University Walk, Bristol, BS8 1TW UK; 2 North Bristol NHS Trust, Bristol, UK; 3 Research Department of Infection & Population Health, UCL, Royal Free Hospital, London, UK; 4 National Institute of Health Research (NIHR) Health Protection Research Unit (HPRU) in Blood Borne and Sexually Transmitted Infections, London, UK; 5 British HIV Association, London, UK; 6 School of Social and Community Medicine, University of Bristol, Bristol, UK; 7 National Institute of Health Research (NIHR) Health Protection Research Unit (HPRU) in Evaluation of Interventions, London, UK

## Abstract

**Background:**

Feedback tools for clinical audit data that compare site-specific results to average performance over all sites can be useful for quality improvement. Proposed tools should be simple and clearly benchmark the site’s performance, so that a relevant action plan can be directly implemented to improve patient care services. We aimed to develop such a tool in order to feedback data to UK HIV clinics participating in the 2015 British HIV Association (BHIVA) audit assessing compliance with the 2011 guidelines for routine investigation and monitoring of adult HIV-1- infected individuals.

**Methods:**

HIV clinic sites were asked to provide data on a random sample of 50–100 adult patients attending for HIV care during 2014 and/or 2015 by completing a self-audit spreadsheet. Outcomes audited included the proportion of patients with recorded resistance testing, viral load monitoring, adherence assessment, medications, hepatitis testing, vaccination management, risk assessments, and sexual health screening. For each outcome we benchmarked the proportion for a specific site against the average performance. We produced performance charts for each site using boxplots for the outcomes. We also used the mean and differences from the mean performance to produce a dashboard for each site. We used principal components analysis to group correlated outcomes and simplify the dashboard.

**Results:**

The 106 sites included in the study provided information on a total of 7768 patients. Outcomes capturing monitoring of treatment of HIV-infection showed high performance across the sites, whereas testing for hepatitis, and risk assessment for cardiovascular disease and smoking, management of flu vaccination, sexual health screening, and cervical cytology for women were very variable across sites. The principal components analysis reduced the original 12 outcomes to four factors that represented HIV care, hepatitis testing, other screening tests, and resistance testing. These provided simplified measures of adherence to guidelines which were presented as a 4 bar dashboard of performance.

**Conclusion:**

Our dashboard performance charts provide easily digestible visual summaries of locally relevant audit data that are benchmarked against the overall mean and can be used to improve feedback to HIV services. Feedback from clinicians indicated that they found these charts acceptable and useful.

**Electronic supplementary material:**

The online version of this article (doi:10.1186/s12913-017-2426-6) contains supplementary material, which is available to authorized users.

## Background

Over the last two decades, HIV organisations and healthcare professionals worldwide have released many sets of clinical guidelines to improve the quality of care that HIV-infected patients receive. In the UK, guidelines for HIV care and treatment are provided by the British HIV Association (BHIVA) and are endorsed by the National Institute for Health and Care Excellence (NICE).

The potential benefits and harms of the existence of clinical guidelines, in general, are discussed by S.H. Woolf et al., 1999 [1]. The main concern about clinical guidelines is their adequacy to cover all individual patients with the same efficiency. Regarding HIV guidelines in particular, the research findings on the disease and its treatment over the years have been systematically implemented to produce evidence-based recommendations in order to maximise benefits and minimise any risks for patients’ health care.

Unfortunately, clinical guidelines themselves do not ensure that a given patient receives the proper care. A commonly used method for assessing the level at which the guidelines are followed by local sites is to conduct an audit survey. An audit, as with all types of evaluation, should be planned in such way that it will give credible information about the outcomes of interest [2, 3]. The feedback from the audit should be used as a tool for quality improvement [4]. Even though some studies have shown that audit and feedback do not have a great effect in general [5, 6], audit can provide an opportunity to improve the quality of professional practice [7].

If the cohort on which the audit is applied involves many sites (clinics), feedback is usually addressed to all participating clinics/bodies in total by publishing a report with the findings of the survey. However, feedback could be more influential if, in addition to the aggregated report, another feedback document were produced for each site individually, which would compare the results of the specific site with the rest of the participating sites. The main concern with implementing this practice is the cost in terms of time and money. The design of a common document template into which audit data could be fed for each site separately could minimise these costs.

A very efficient method commonly used for the provision of feedback to clinics when benchmarking of performance is required, is the use of performance charts. There are several ways of constructing such charts, depending on the information to be represented. Some of the advantages of performance charts compared to written reports are that, if designed properly, they are easy to read, convey much information, and are appropriate for comparison purposes [8, 9]. A disadvantage of this method is that if there are many items of data, charts may become too busy. Moreover, if items are strongly correlated then there will be redundancy in the information presented and trends in performance of different aspects of care may be more difficult to define as many indicators will show similar performance. A more useful approach could be to aggregate correlated items to minimise the potential repetitiveness of the feedback and avoid redundancy.

In 2015, BHIVA conducted a national audit survey in order to assess the compliance of UK HIV health clinics with the 2011 BHIVA guidelines for routine investigation and monitoring of adult HIV-1- infected individuals [10]. The survey collected data on a number of outcomes, described in detail in the methods section of this paper, for which the sites had specific guidelines. The audit report derived from the survey [11] gives a detailed analysis of the aggregated performance of the participating sites, comparing the results with the target values set for the outcomes.

The aim of this article is to propose a feedback tool that can be sent to each of the participating HIV clinics and adapted for other clinical situations, which can be used for the quality improvement of the aspects of care where the site seems to underperform compared to the other sites. The proposed tool should give a clear indication of the benchmark of the site’s performance, so that a relevant action plan can be directly implemented in order to improve the care services the site provides to the patients. Using the BHIVA audit data we developed charts that show the performance of each site relative to the distribution of performance in all sites, and dashboards which benchmark to the mean performance and show difference of each site’s performance from the mean. We then accounted for correlations between aspects of HIV care in order to produce simplified charts for use by HIV health care providers.

## Methods

### Data collection

The data were collected between June–August 2015 as BHIVA’s annual audit project for that year. UK clinic services providing specialist HIV care were invited to provide data on a sample of 50–100 patients by completing a self-audit spreadsheet [12]. The spreadsheet included a list of random numbers which clinics were asked to match against a de-duplicated list of all adult patients (aged 16 or over) seen for HIV care during 2014 and/or 2015 (e.g. as prepared for public health surveillance reporting) to generate a random sample.

The outcomes we focussed on are described in Table 1, where the dichotomization of the outcomes is also explained by clarifying what is considered to be the positive case for each outcome. The last column of the table states the inclusion criteria for patients for each outcome.

**Table 1:** Outcomes assessed in the 2015 BHIVA Audit with inclusion criteria for patients

Outcome	Explanation of positive outcome	Inclusion criteria for patients
Resistance done	Resistance test done and/or sample stored	All
VL measured	Viral load (VL) measured within past 6 months	Patients on ART
Adherence assessed	Adherence assessed within past year	Patients on ART
Medications recorded	All medication recorded within past year	Patients on ART
Hep A immune	Vaccinated or otherwise immune to hepatitis A	All
HBsAg known	Hepatitis B surface antigen (HBsAg) status is known	All
Hep C tested	Hepatitis C (Hep C) antibody status is known	All
CVD risk assessed	Cardiovascular disease (CVD) risk assessed, within past 3 years if on ART, ever if not on ART	All
Smoking assessed	Smoking status recorded within past two years	All
Flu vaccination managed	Flu vaccination recorded as done, or recorded as advised to obtain from General Practice (GP) within past year	All
SH screening offered	Sexual health (SH) screening offered within past year	All
Cervical cytology managed	Cervical cytology recorded as done, or recorded as advised to obtain elsewhere, within past year	Females
BMD measured	Bone mineral density (BMD) measured	Age > 70 and on ART
FRAX risk assessed	Fracture risk assessed within past 3 years	Age > 50
Pneumococcus vaccinated	Vaccinated against pneumococcus	CD4 > 200 cells/mL

*ART* antiretroviral therapy

### Statistical analysis

#### Summary statistics

The software used for the following statistical analysis and the proposed graphical methods for performance benchmarking, was StataMP 14 [13].

For each of the 15 outcomes, we tabulated the number of sites contributing data, the total number of patients audited, and the mean, standard deviation, minimum and maximum proportion of patients with a positive outcome across all sites. To show summary statistics on the proportion of positive cases for each outcome, we plotted boxplots for the outcomes over all sites. The median is shown inside the box which extends from the 25th to 75th percentile, the whiskers show the central 95% of the distribution and outliers are shown as separate points. Note that for some outcomes the distribution is truncated at the upper end because sites achieved 100% positive cases.

#### Site-specific statistics used to create performance charts

To benchmark the proportion of positive cases for a specific site against the average performance, for each site separately we superimposed the site’s estimated proportion (marked by a white triangle) with 95% confidence interval onto the boxplot showing the overall summary statistics. The site’s proportion for each outcome was estimated by the proportion of patients in the sample with the positive outcome as described in Table 2. The 95% confidence interval was calculated for this point estimate. We produced performance charts with boxplots for each outcome in the audit for each site.

**Table 2:** Summary statistics on the proportion of positive outcomes for all sites

Outcome (BHIVA 2015 audit)	Number of sites	Number of patients	Mean (of sites)	St. dev	Min	Max
Resistance done	106	7768	81.0%	12%	42.0%	100.0%
Viral load measured	106	6978	89.5%	10%	32.6%	100.0%
Adherence assessed	106	6978	93.7%	9%	45.5%	100.0%
Medications recorded	106	6978	89.4%	12%	44.7%	100.0%
Hep A immune	106	7768	60.3%	28%	0.0%	100.0%
HBsAg known	106	7768	92.5%	16%	0.0%	100.0%
Hep C tested	106	7768	96.3%	6%	52.0%	100.0%
CVD risk assessed	106	7768	43.6%	28%	0.0%	100.0%
Smoking assessed	106	7768	65.9%	26%	0.0%	100.0%
Flu vaccination managed	106	7768	55.3%	33%	0.0%	100.0%
Sexual health screening offered	106	7768	64.8%	23%	6.0%	98.1%
Cervical cytology managed	106	2663	74.6%	18%	9.1%	100.0%
Bone mineral density measured	70	150	17.4%	31%	0.0%	100.0%
FRAX risk assessed	106	2413	17.9%	24%	0.0%	93.8%
Pneumococcus vaccinated	106	7411	25.1%	31%	0.0%	100.0%

*CVD* cardiovascular disease; Hep hepatitis; HBsAg hepatitis B surface antigen

#### Presentation of site-specific data using benchmarked dashboards

**Fig. 1:**
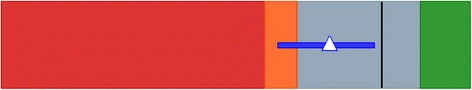
Explanation of dashboard coloured areas which show differences from the overall mean. Legend: The *black* vertical line shows the overall proportion (mean) for all sites. The coloured areas indicate the performance of the site. *Grey* area corresponds to values between 90%–110% of the overall mean (sites falling in this area are considered to perform as expected). *Green* area corresponds to values >110% of the overall mean (sites falling in this area are considered to perform better than expected). *Orange* area corresponds to values between 80%–90% of the overall mean (sites falling in this area are considered to perform worse than expected). *Red* area corresponds to values <80% of the overall mean (sites falling in this area are considered to perform much worse than expected). The white triangle shows the estimated proportion (score) for the site in question and the blue bar gives the 95% confidence interval for the site’s proportion. In this graph, the estimated score falls within the *grey* area, so there is no strong evidence of bad performance

In addition to using box plots based on the median and the distribution of positive outcomes across all sites to benchmark each site’s performance, we also used dashboards based on the mean values. In particular, a template dashboard for each outcome was produced, on which was marked the overall mean (marked as a vertical black bar) and coloured bands representing difference in performance from the mean (Fig. 1). We defined performance for each outcome separately based on the following percentage cut-offs relative to the mean: better than expected (>110% green), as expected (90–110% grey), worse than expected (80–90% orange), and much worse than expected (<80% red) performance (Fig. 1). Then, in a similar way to the performance charts, the individual site estimated proportion (white triangle) and 95% confidence interval (horizontal blue bar) were positioned on the template to show the benchmarked performance for the outcome.

#### Data reduction using principal component analysis – Simplified performance charts

We estimated correlations between outcomes to see if outcomes could be grouped. Some of the outcomes were clearly related, for example, the three outcomes on hepatitis A, B and C, therefore we used principal components analysis (PCA) to reduce the number of data items. The PCA was done using the rankings of the site proportions, instead of the proportions themselves, in order to eliminate the difference in variability between the outcomes. If a site had the highest proportion for an outcome then it would be ranked 1st, the second highest would be ranked 2nd and so on. Where there were equalities, the average rank was assigned. Then, 5 groups of rankings were formed according to quintiles of the sites (1–21, 22–42, 43–63, 64–84 and 85–106). Hence, for each of the outcomes, every site would lie in one of the 5 ranking groups. The groups were formed because for some outcomes the distribution of values was very dense. Hence, a big difference in ranking did not necessarily correspond to a big difference in the actual proportion of positive cases. In the PCA, we retained factors if their eigenvalue was >1 and used loading size (nominally using threshold of 0.4) to allocate outcomes to factors. The results of the rotated PCA indicated four factors could represent the outcomes. Hence, for each of the factors we created a score by taking the unweighted average of the proportions of positive cases of the corresponding outcomes included in the factor. We then produced performance charts for each site with the number of boxplots or dashboards reduced to four using the same groupings of outcomes derived from PCA.

Analyses were conducted in part through the National Institute for Health Research Units (NIHR HPRUs) in Evaluation of Interventions (University of Bristol) and Blood Borne and Sexually Transmitted Infections (University College London in collaboration with London School of Hygiene and Tropical Medicine), both of which are in partnership with Public Health England.

## Results

### Summary statistics

A completed self-audit spreadsheet was returned by 123 clinic sites, of which 17 were excluded because they provided data on fewer than 40 patients. 16 (94%) of 17 excluded sites were outside London, as compared with 89 (85%) of 106 included sites. A sensitivity analysis was performed to test whether removal of these sites would result in significant differences in the overall mean of each of the outcomes. Results showed that there were no systematic differences. Removal of the 17 clinics resulted only in small differences: the largest observed for the CVD risk assessed outcome (+1.7%) and the Smoking assessed outcome (−1.1%) (see, in Additional file 1: Table S1).

**Fig. 2:**
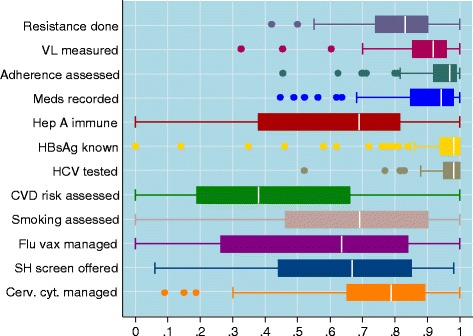
Boxplots showing the distribution of site performances for each outcome. Legend: The main body of a boxplot (coloured box) covers the range between the lower and upper quartiles. The white vertical line shows the position of the mean. The lines (*whiskers*) outside the boxes are extended as to cover all data points that lie within ±1.5 IQR (interquartile range, which is the difference between the upper and lower quartiles) form the upper and lower quartiles respectively. The dots indicate the points falling beyond the whiskers and they are usually considered to be outliers (the boxplot were constructed in STATA. A more detailed analysis of boxplot can be found in [20])

The 106 sites included in the study provided information on a total of 7768 patients. A summary of the proportion of positive cases for each outcome is given in Table 2 and their distribution is illustrated in Fig. 2 using a boxplot showing their variability. The first four outcomes which capture monitoring of treatment of HIV-infection show high performance across the sites, whereas testing for hepatitis A and B appears not to be done at all in some sites. Assessments of cardiovascular disease (CVD) risk and smoking, management of flu vaccination, sexual health screening, and cervical cytology for women are also very variable across sites. The last three outcomes, measuring bone mineral density, assessing fracture risk, and offering pneumococcal vaccination show very low overall performance. Despite accounting for eligibility of patients, the audit shows that recommended guidelines are poorly adhered to for these three outcomes. The audit recommended implementing an action plan for these three outcomes across all sites and therefore we excluded them from further comparisons between sites, thus the following analysis includes only the first 12 outcomes. A more detailed analysis of the outcomes and the target values for each of them has been published elsewhere [11].

#### Site-specific performance charts and dashboards

**Fig. 3:**
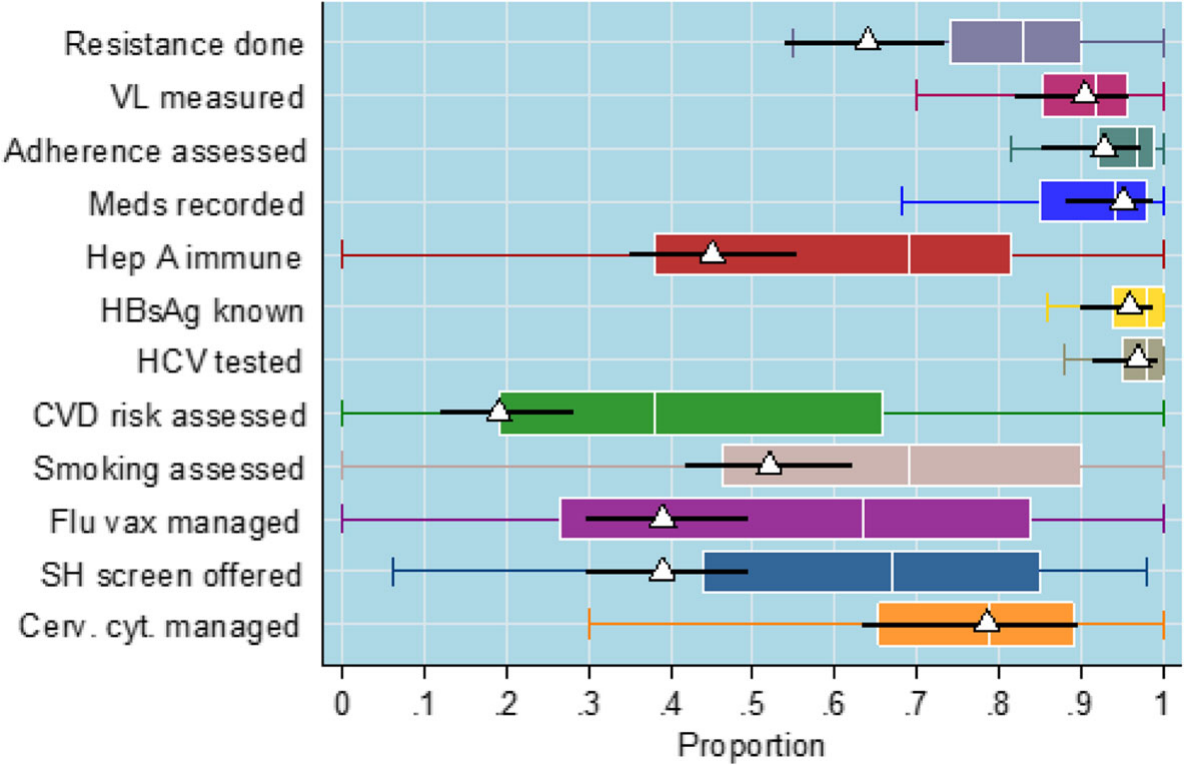
Example of Boxplots-Performance Chart - result for example site. Legend: The boxplots are the same as in Fig. 2 except that the outliers here are removed for clarity. The estimated scores of the site, for each outcome, are represented by the white triangles. The horizontal black lines give the 95% confidence intervals for the site’s scores

An example of a performance chart is shown in Fig. 3 which compares the performance of site 1 with the performance of all sites. This uses the boxplot showing the distribution of site performances (Fig. 2 minus the outliers for clarity) as a template and superimposes the mean and 95% confidence intervals for site 1 for each outcome. The chart shows analytically (for each outcome) where the score of the site is ranked in comparison with the other sites. Form the graph it is clear that site 1 performs relatively badly for resistance testing and sexual health screening outcomes (although it is not an outlier), but performs better than 50% of the sites for recording medications and is within the interquartile range for all other outcomes.

**Fig. 4:**
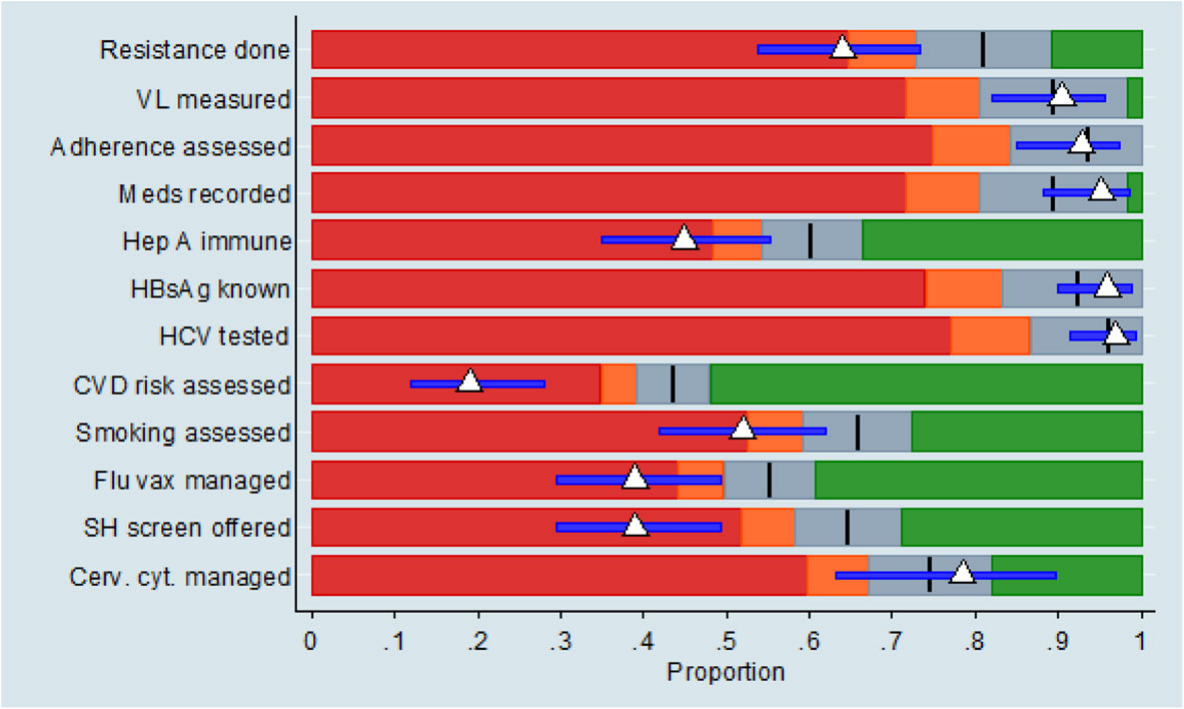
Example of Dashboard showing benchmarked performance for example site. Legend: The colourings on the chart are explained analytically in Fig. 1. For the specific site, we observe that the scores and 95% CI for outcomes SH screening offered and CVD risk assessed, fall completely within the red areas, indicating a very poor performance. A similar situation is observed for Resistance done, Hep A immune, Smoking assessed and Flu vax managed where the estimate proportions fall within the red area, however the CI indicate that the true proportions could be less worrying. For all other outcomes the results are as expected

The alternative representation of performance using the dashboard to show the percentage difference between the site-specific performance and the mean performance across all sites is shown for site 1 in Fig. 4. This representation shows that 6 out of the 12 outcomes are in the red band denoting much worse performance than average. Two outcomes, CVD risk assessment and sexual health screening, have their entire 95% confidence interval in the red section, showing that poor performance cannot be attributed to sampling variation.

#### Data reduction using PCA – Simplified performance charts

In the PCA there were four factors with eigenvalues >1 which were retained in the model (in Additional file 1: Table S2). These results showed that we may reduce the number of outcomes by replacing them with four representative factors. The loadings for the factors (in Additional file 1: Table S3) were used to identify the factors and allocate outcomes to them. The proposed factors were:

- Factor 1 (**HIV Care**): Viral load done, adherence assessed, medications recorded.
- Factor 2 (**Hepatitis testing**): Hep A immune, HBsAg known, Hep C tested.
- Factor 3 (**Other Screening tests**): CVD risk assessed, Smoking assessed, Flu vaccination managed, sexual health screening offered, cervical cytology managed.
- Factor 4: Resistance testing.

**Fig. 5:**
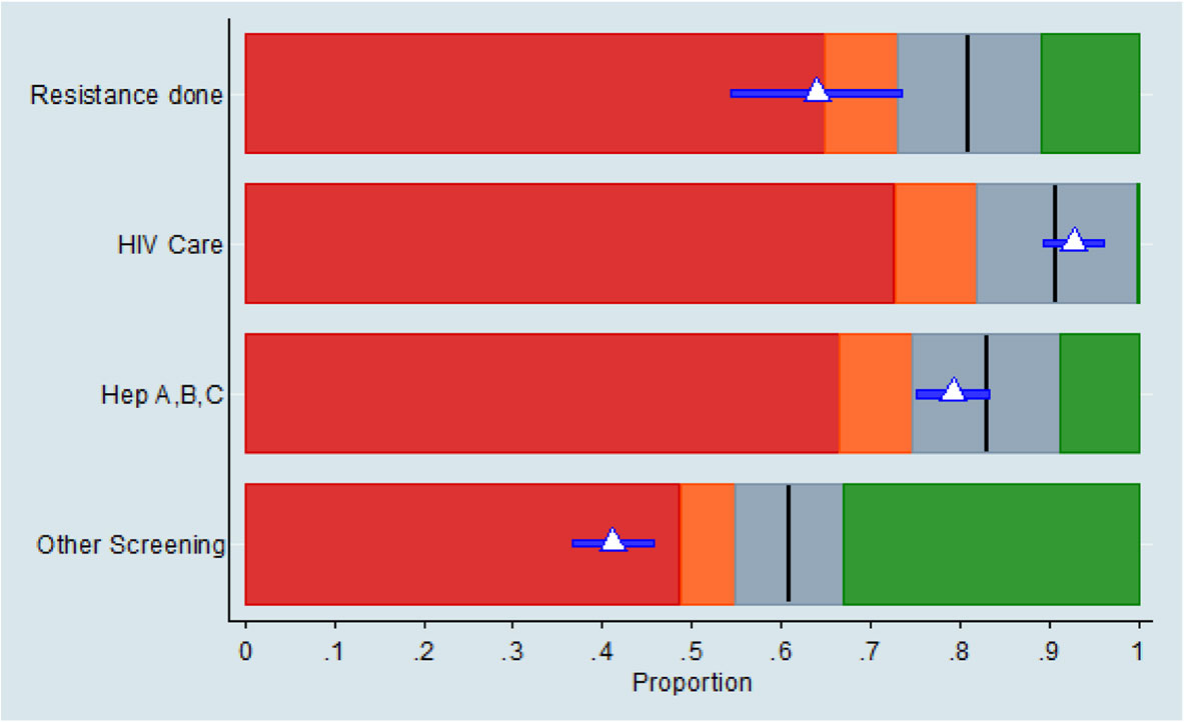
Dashboard using the four factors showing benchmarked performance for example site. Legend: The PCA suggests the use of 4 factors instead of the 12 outcomes. So, for the example site (same as the one used for Fig. 4) the performance for HIV care and Hep A, B, C is as expected. On the other hand the performance for Resistance done (this is the same as in Fig. 4) and especially Other Screening is much worse than expected

We allocated the outcome cervical cytology management to factor 3 even though it had larger loadings for factors 1 and 2 as clinically it is more reasonable to group it with the screening outcomes in factor 3. The simplified dashboard for site 1 is shown in Fig. 5 based on the four factors derived from the 12 original outcomes. Even though the information for each outcome separately is no longer available from the simplified dashboard, it is now easier to read the results and have a clearer idea on how the clinic is performing compared to other clinics in each of the four aspects of clinical services for HIV. In particular, the example clinic showed adequate performance in HIV care and Hepatitis testing, whilst resistance testing and other screening were less frequently performed than in other clinics (Fig. 5).

## Discussion

Using the 2015 BHIVA audit data, we have proposed two different ways of visually presenting clinics with site-specific audit outcomes benchmarked against the corresponding overall average outcomes.

For the specific outcomes, the overall average of the outcomes was preferred as a criterion for assessing the performance of the clinics, instead of using target values suggested by clinical guidelines. The reason is that most of these target values, if they exist, are not evidence-based and they are usually arbitrarily set by what experts believe that clinical services ought to be able to achieve. Thus, comparison with the actual mean performance was considered a more valid approach. However, in cases where the target values of clinical guidelines are the primary aim of an audit, the proposed methodology could still be applied with slight modifications.

Both the boxplot and the dashboard performance charts give an immediate impression of how a particular clinic is performing compared with the average over all clinics. The boxplot chart locates the clinic position relative to the ranking of all clinic sites, whereas the dashboard illustrates an absolute measure of performance in bands centred on the mean performance.

The first performance charts we constructed showed information for all of the audit outcomes, however, participating clinics required an easily interpretable summary of the results which was best represented by less detailed graphs. We have shown the feasibility of using PCA to group similar outcomes and simplify the format of the performance charts by reducing the number of boxplots or dashboards. Represented in this way, with grouping of correlated variables, it is much easier to assess where to direct change. The proposed performance charts can be used to inform HIV care and improve service delivery in a locally relevant way. Acceptability and usefulness of the dashboards were assessed via a well-attended open forum at BHIVA conference in April 2016 where we presented different ways of visualising outcomes data and facilitated clinicians in discussing the merits of each. While this forum did not have the rigour of a formal focus group, it did indicate that clinicians preferred the simplified dashboard format and judged it to be useful. In feeding back to clinical services we did provide them with their data for each individual indicator as well as the clustered factors shown in the dashboard. Furthermore, similar methods could be adapted for use in audits of other clinical services.

Whilst in general the standard of HIV care in the UK is considered to be very good [14], the audit does highlight gaps in adherence to guideline recommendations, particularly with respect to screening and risk assessment for co-morbid conditions commonly found in people living with HIV. These gaps may be more likely to be addressed when highlighted at the local level as we have tried to do with these site-specific performance charts. Although only some of the clinics may be able to offer a full range of services on their own site, all clinics can adhere to guidelines by appropriately referring patients to other providers of such services. For example, patients may need to see their General Practitioner (GP) in order to be vaccinated against influenza. It is likely in the future that HIV services will become more constrained to deliver only treatment and care for a patient’s HIV infection. This will make it even more important that patients are correctly signposted to primary and secondary care services that guidelines recommend they receive.

### Context

BHIVA has conducted audits since 2001, mainly by asking clinicians to retrospectively review individual patient case-notes, as in this instance. The aim of its audit programme is to assess how well routinely delivered adult HIV care adheres to existing standards and guidelines, so as to identify areas for quality improvement at national and individual service-provider levels.

The audit complements research carried out by the UK Collaborative HIV Cohort (UK CHIC). Observational cohorts such as UK CHIC focus on patient outcomes. These include biomarkers for assessing treatment response and patient health, such as viral load and the CD4 count, and clinical AIDS disease and mortality, but also monitoring of, for example, renal function, liver enzymes, cholesterol and blood pressure. Audit outcomes focus on processes and whether these have been carried out according to guidelines. For example, the audit asks if the CD4 count has been measured, whereas a research study would analyse the values of CD4 count attained by patients. The BHIVA audit informs on whether appropriate care has been offered to patients, but not on how well patients do. For example, a screening test can be offered to a patient, but the patient can refuse the offer and therefore not benefit. The use of the term audit is not always restricted to whether processes are followed, although it is in our study example.

An interesting question is whether audit data should be adjusted for patient demographics or case-mix. Recent studies using data from UK CHIC found that differences between clinics in the proportion of patients with (i) low CD4 at presentation to care and (ii) viral suppression 1 year after starting ART were explained by patient mix [15, 16]. However, the outcomes in the BHIVA audit appeared to be independent of patient mix and therefore did not need to be adjusted (for further explanation of the adjustment method used and results see Additional file 2). As you would expect, viral load testing offered by a clinician is independent of patient demographics, whereas viral suppression, which depends on patient adherence to ART, may depend on sex, age, ethnicity [17, 18] and transmission risk group. Nevertheless, it is important to check independence for each outcome as, for example, the offer of hepatitis testing could depend on perceptions of the clinician about how much ‘at risk’ of hepatitis the patient is.

#### Other plots for benchmarking and their use

Alternative plots used to benchmark site performances include caterpillar and funnel plots. Caterpillar plots display in rank order the point estimate of an outcome with 95% confidence interval for every site. These have been criticised [19], particularly in the field of education where they represent league tables for schools based on pupils’ exam achievements, as the order can be very variable from year to year and the extreme estimates may still be within the bounds of an expected distribution. If it is more important to identify outliers then a funnel plot [20] is often used [19]. The proportion of the outcome (or the ratio of observed to expected) is plotted against site size. A horizontal line is drawn at the population mean (or at 1) and funnels are drawn to represent 95% (and/or 99%) confidence limits for the sample means according to site size. Site estimates of the outcome are then plotted against site size and those that fall outside the funnel shape are outliers. Funnel plots can also be drawn centred on absolute targets rather than the population mean. However, these plots require complete data on all patients if they are to be unbiased. They are not suitable for audits based on random sampling such as our study.

#### Limitations

The BHIVA audit is a sample of HIV patients attending each clinic and therefore is not as complete as audits undertaken in some disease areas where registries exist. For example, the Renal Registry collects data on all patients starting dialysis in the UK [21]. The BHIVA audit committee requested random sampling of patients attending clinic throughout the year, but included patients may have been biased towards those attending clinics at the time of the audit who may be more likely to be new patients or patients with greater healthcare needs. Clinics who submitted less than half the patients requested were omitted from these analyses because we thought that there might be selection bias. Of note, clinic size was not associated with sample size. Because the BHIVA audit is topic-based and does not collect data on the same outcomes each year, its impact in terms of improving performance of service delivery is difficult to judge. However, improvements may be more likely if locally relevant feedback is given in a clear format which is easily understood, in addition to receiving reports of aggregated data. The main concern with implementing this practice is the cost in terms of time and money. Nevertheless, the use of the audit data and the design of a common document template for all sites could minimise these costs.

#### Future recommendations

It will be important to measure the impact on service improvement of using performance charts to feedback clinic-specific benchmarked audit data. This will require repeat collection of data to assess trends over time. Incorporating quality control methods for outliers would allow clinics to compare their data with previous years and take rapid action in case of deteriorating outcomes. As hospitals improve capture of such data, regular audit will become more feasible.

## Conclusion

The BHIVA clinical audit is part of a quality improvement cycle that involves measurement of the delivery of healthcare for people living with HIV against agreed evidence-based standards for high quality. In order for clinicians to take action to bring practice in line with these standards and so improve the quality of care and health outcomes, it is necessary to feedback the results of the audit in a meaningful and impactful format [6]. Based on the BHIVA audit, we have proposed easily digestible visual summaries of locally relevant audit data that are benchmarked against the overall mean which can be used for improving HIV services.

We have shown that principal components analysis can be used to simplify the representation of multiple correlated audited variables. The aggregated data is more meaningful when comparing correlated data and allows both accurate benchmarking against the average performance and against a standard, either of which can be used to measure future improvements.

## Additional files

Additional file 1: **Table S1.** Results for all 123 clinics - Sensitivity analysis for the removal of the 17 sites. **Table S2.** Principal Components Analysis - suggested factors (eigenvalue > 1). **Table S3**. Principal Components Analysis – factor loadings. (DOCX 17 kb)

Additional file 2: Adjustment for patient mix. (DOCX 114 kb)

## Abbreviations

ART: Antiretroviral therapy
BHIVA: British HIV Association
BMD: Bone Mineral Density
CI: Confidence Interval
CVD: Cardiovascular Disease
GP: General Practice/Practitioner
HBsAg: Hepatitis B surface Antigen
Hep: Hepatitis
HIV: Human Immunodeficiency Virus
HPRU: Health Protection Unit
IQR: Interquartile Range
NICE: National Institute of Care Excellence
NIHR: National Institute of Health Research
PCA: Principal Component Analysis
PHE: Public Health England
SH: Sexual Health
UK CHIC UK: Collaborative HIV Cohort
VL: Viral Load
