# Supplementary material for: Benchmarked performance charts using principal components analysis to improve the effectiveness of feedback for audit data in HIV care
Source: BMC Health Serv Res. 2017 Jul 24;17:506. doi: 10.1186/s12913-017-2426-6 (PMC5525257; doi:10.1186/s12913-017-2426-6)
Supplement: Supplementary file 1 — Table S1. Results for all 123 clinics - Sensitivity analysis for the removal of the 17 sites. Table S2. Principal Components Analysis - suggested factors (eigenvalue > 1). Table S3. Principal Components Analysis – factor loadings. (DOCX 17 kb) [file 12913_2017_2426_MOESM1_ESM.docx]

**Supplementary Tables**

Supplementary Table 1

Results for all 123 clinics - Sensitivity analysis for the removal of the 17 sites

| **Outcome (BHIVA audit 2015)** | **Number of sites** | **Number of patients** | **Mean (of sites)** | **Std. Dev. (of sites)** | **Min** | **Max** |
| --- | --- | --- | --- | --- | --- | --- |
| Resistance done | 123 | 8,258 | 81.5% | 12.4% | 42.0% | 100.0% |
| Viral load measured | 122 | 7,395 | 90.1% | 10.1% | 32.6% | 100.0% |
| Adherence assessed | 122 | 7,395 | 93.7% | 9.1% | 45.5% | 100.0% |
| Medications recorded | 122 | 7,395 | 89.2% | 12.9% | 40.0% | 100.0% |
| Hep A immune | 123 | 8,258 | 59.9% | 28.7% | 0.0% | 100.0% |
| HBsAg known | 123 | 8,258 | 92.5% | 15.8% | 0.0% | 100.0% |
| Hep C tested | 123 | 8,258 | 96.3% | 6.0% | 52.0% | 100.0% |
| CVD risk assessed | 123 | 8,258 | 41.9% | 28.8% | 0.0% | 100.0% |
| Smoking assessed | 123 | 8,258 | 67.1% | 26.2% | 0.0% | 100.0% |
| Flu vaccination managed | 123 | 8,258 | 56.1% | 32.9% | 0.0% | 100.0% |
| Sexual health screen offered | 123 | 8,258 | 65.6% | 22.9% | 6.0% | 100.0% |
| Cervical cytology managed | 122 | 2,763 | 74.0% | 20.9% | 0.0% | 100.0% |
| Bone mineral density measured | 78 | 167 | 16.4% | 29.9% | 0.0% | 100.0% |
| FRAX risk assessed | 123 | 2,568 | 16.8% | 22.9% | 0.0% | 93.8% |
| Pneumococcus vaccinated | 123 | 7,877 | 25.4% | 30.2% | 0.0% | 100.0% |

Table gives results for outcomes before the exclusion of the 17 sites. No big differences observed compared to Table 2 of main article.

Supplementary Table 2

PCA- Suggested factors (eigenvalue>1)

| Factor analysis/correlation | | | Number of obs=106 | | |
| --- | --- | --- | --- | --- | --- |
| **Method**: principal-component factors | | | Retained factors=4 | | |
| **Rotation**: orthogonal varimax (Kaiser off) | | | Number of params=42 | | |
|  | | |  | | |
| **Factor** | **Eigenvalue** | **Difference** | | **Proportion** | **Cumulative** |
| Factor1 | 2.360 | 0.331 | | 19.67% | 19.67% |
| Factor2 | 2.029 | 0.079 | | 16.91% | 36.58% |
| Factor3 | 1.950 | 0.845 | | 16.25% | 52.83% |
| Factor4 | 1.105 | . | | 9.21% | 62.04% |

Supplementary Table 3

PCA - Loadings for the 4 factors

| **Outcome (grouped rankings)** | **Factor 1**  **HIV care** | **Factor 2**  **Hepatitis testing** | **Factor 3**  **Other screening tests** | **Factor 4 Resistance testing** | **Uniqueness** |
| --- | --- | --- | --- | --- | --- |
| Resistance done | 0.056 | 0.075 | -0.036 | **0.952** | 8.32% |
| VL measured | **0.699** | 0.177 | -0.168 | -0.218 | 40.38% |
| Adherence assessed | **0.811** | -0.005 | 0.203 | 0.218 | 25.37% |
| Meds recorded | **0.726** | 0.071 | 0.211 | 0.095 | 41.44% |
| Hep A immune | -0.197 | **0.686** | 0.112 | 0.036 | 47.64% |
| HBsAg known | 0.134 | **0.724** | 0.211 | 0.255 | 34.75% |
| HCV tested | 0.203 | **0.840** | 0.061 | -0.024 | 24.82% |
| CVD risk assessed | 0.045 | 0.077 | **0.800** | -0.017 | 35.26% |
| Smoking assessed | 0.142 | 0.171 | **0.775** | -0.036 | 34.90% |
| Flu vax managed | 0.364 | 0.299 | **0.427** | 0.129 | 57.93% |
| SH screen offered | 0.362 | 0.143 | **0.534** | -0.035 | 56.27% |
| Cerv. cyt. managed | 0.547 | 0.375 | **0.260** | -0.090 | 48.45% |

*Bold cells indicate the factor into which the corresponding outcome is incorporated. Note that for the last outcome, the factor selected is not the one with the largest loading, but the one that is more meaningful.*
