# Supplementary material for: Benchmarked performance charts using principal components analysis to improve the effectiveness of feedback for audit data in HIV care
Source: BMC Health Serv Res. 2017 Jul 24;17:506. doi: 10.1186/s12913-017-2426-6 (PMC5525257; doi:10.1186/s12913-017-2426-6)
Supplement: Supplementary file 2 — Adjustment for patient mix. (DOCX 114 kb) [file 12913_2017_2426_MOESM2_ESM.docx]

**Additional file 2**

**Adjustment for Patient Mix**

The method used to test the patient mix effect in the outcomes is the following: We first used a logistic regression model for each outcome, with covariates the age and the combined variable sex/ethnicity/exposure of the patients. Hence, we obtained the expected percentage of positive outcome for each clinic. We plotted the adjusted ratios, which is the observed proportion (OP): expected proportion (EP). We produced the same ratios for the unadjusted case (i.e. considering that all sites are expected to have the same mean, independently of their patient mix). Comparing the two graphs, it was clear that there were no substantial differences, indicating that that patient mix does not affect the results for the outcomes of BHIVA audit. In particular, observing the movement of the lowest points on the left graph (i.e. those indicating a much lower proportion than overall proportion) after the adjustment, there is no sign of patient mix effect on the outcomes.

Below there are some examples, illustrating the independence of BHIVA audit outcomes with patient mix. (First graph: Resistance, Second graph: Viral load, Third graph: HBsAg, Fourth graph: Sexual Health screening). Left graphs show the ratios of OP: EP without any adjustment for each of the participating sites. Right graphs show the ratios of OP: EP for each site, with adjustment for patient mix, where the adjustment is applied using logistic regression with covariates the sex, age ethnicity and exposure of patients
